# Supplementary material for: The Pathways to Participation (P2P) Program: A Pilot Outcomes Study
Source: Int J Environ Res Public Health. 2022 May 17;19(10):6088. doi: 10.3390/ijerph19106088 (PMC9141954; doi:10.3390/ijerph19106088)
Supplement: Supplementary file 1 [file ijerph-19-06088-s001.zip › ijerph-1665566-supplementary.pdf]

International Journal of
Environmental Research and Public Health

MDPI

Pathways to Participation (P2P) program

Session Plan

Week 1

Equipment: P2P workbook, post its/ butchers' paper, pens/pencils/markers

1. Begin the session with an ice-breaker game (facilitators choice). (5-10 minutes)

2. Introduce P2P program including what will be covered over the 10 weeks. Conclude with brief overview of today's activities. (10-15 minutes)

3. Formulate group expectations via a group discussion. (20 minutes)

   Suggested approaches:

   Provide post it notes and ask consumers to anonymously write an expectation they believe to be important. Place these on a wall or whiteboard and facilitate group discussion.

   Use butcher's paper have an open group discussion and brainstorm about what both facilitators and consumers would like to have as group expectations. This can be kept on display at later sessions.

TEA BREAK. (15 minutes)

4. Complete "Self Profile" (Worksheet 1.1). Consumer tick the statements most applicable to their own situation. Those which remain unticked may form the basis of goals and action plans. (40 minutes)

5. Introduce Homework sheet 1 (Daily Time Use Diary). Explain the task, which involves completing the table to describe activity patterns over a 24hr time block on a typical day within their week.

   Suggested approach:

   Provide an example diary entry

6. Questions and Feedback. Provide post it notes (as exit slips) to everyone and ask consumers to write the three things they will take away from this session. The points may be questions, good aspects, or things to change. Emphasise to consumers they do not need to put their names on the slips. These are deposited in a box as consumers leave. As consumers are completing these slips, provide a brief overview of the next session. (10 minutes)

Week 2

Equipment: P2P workbook, post its/ butchers' paper, pens/pencils/markers, completed time diary

1. Overview of the session: Begin by inviting any reflections / feedback from the previous session before providing a brief overview of todays' activities. (5 minutes)

2. Complete "Considering the balance of my activities" (Worksheet 2.1): Consumers to use the responses from their time use diary to complete worksheet, entering examples of activities they completed into each category (self-care, productive, leisure and rest). Consumers then add up the total time spent completing activities of each category and are supported to convert this into a graph using a template provided. (30 minutes)

   Suggested approach:

   If time diary was not complete for homework (or new participants join group), ask consumers to reflect on the last 24 hours and base the workshop on these activities.

3. Complete "Finding meaning in my activities" (Worksheet 2.2) while facilitating a group discussion: Consumers reflect on statements around the topic of "I would like to participate in activities that…", and tick the box which most closely matches their response to each statement. (10 minutes)

4. Complete "My satisfaction with my activities" (Worksheet 2.3) while facilitating a group discussion: Consumers write or draw a picture in response to 7 questions around activities they find enjoyable, not enjoyable or distressing. (20 minutes)

   Alternative: Consumers can go through their time use dairy and draw happy or sad faces next to their activities, depending on whether they are currently satisfied or not.

   TEA BREAK. (15 minutes)

5. Complete "Social interactions through activities & accessing my community" (Worksheet 2.4) while facilitating a group discussion: Consumers name people they see regularly or on a typical day, identify the relationship they have with them, and describe the activities they would typically complete together. (15 minutes)

6. Complete "Accessing my community" (Worksheet 2.5): Group (or pair) discussion of places they visit regularly in their local community, before a combined list is drawn up from everyone's responses. The worksheet also poses some brief questions relating to time they spend at home vs the community. (15 minutes)

7. Complete "Activity Engagement Measure" (Worksheet 2.6): Consumers reflect on 7 brief statements relating to this week's content and circle a response that reflects how true they find the statement. Lead a brief group discussion on the responses people are willing to share. (10 minutes)

8. Questions and Feedback. Repeat from previous week

Week 3

<u>Equipment:</u> P2P workbook, post its/ butchers' paper, pens/pencils/markers

1. Overview of the session: Repeat from previous week. (5 minutes)

2. Complete "Activity patterns I would like to change" (Worksheet 3.1): Given the information gathered in Week 2, what area would each consumer like to see change over the next few weeks? Each consumer completes activity independently and are encouraged to keep these goals in mind for the activities later in this session (15 minutes)

3. Complete "Benefits of my current activities" (Worksheet 3.2) while facilitating a group discussion: Consumers read through the listed statements, and then indicate whether they are applicable to their life. (10 minutes)

4. Complete "Personal qualities and traits" (Worksheet 3.3) while facilitating a group discussion: Consumers encouraged to think about their positive qualities by circling examples or providing their own. Discussion should focus on how to tell others about these qualities, using examples and/or role play. Combined list of qualities can be placed on the board to illustrate the groups collective strengths. (30 minutes)

TEA BREAK. (15 minutes)

5. Complete "What skills do I have?" (Worksheet 3.4) while facilitating a group discussion: Consumers tick the skills they already have and identify examples of when they've displayed it. (15 minutes)

6. Complete "My positive self" (Worksheet 3.5): Consumers respond to a series of statements that provides further examples of telling people about their strengths and skills. This activity may be quite challenging for some consumers, and they choose to complete it as homework. (20 minutes)

7. Questions and Feedback. Repeat from previous week

Week 4

<u>Equipment:</u> P2P workbook, post its/ butchers' paper, pens/pencils/markers

1. Overview of the session: Repeat from previous week. (5 minutes)

2. Group discussion about stress: Why does it happen? What is good stress (eustress) versus bad stress? Why is it important to manage stress? What are the consequences of prolonged stress on health (physical, mental, emotional). (15 minutes)

3. Complete "Reducing stress in activity participation" (Worksheet 4.1): Consumer review a list of common stress reducing strategies and identify what they currently use and which they want to try. Facilitate a group discussion about how people currently manage stress and encourage the group to

brainstorm other strategies that consumers find effective in managing stress. (20 minutes)

TEA BREAK. (15 minutes)

4. Complete “The pros and cons of my current activity patterns” (Worksheet 4.2): Facilitate group discussion about activities completed in the categories of self-care, productivity, leisure and rest. For each identified, ask the consumers to think of an example of how this activity might improve their health and wellbeing (considering physical, mental, spiritual and emotional dimensions of health). Then, ask consumers to think of examples of how this activity might have a detrimental impact on their health (e.g., sleep is important for the immune system and much more, BUT sleeping too much during the day loses productive time and increases likelihood of weight gain). 30 minutes

   Suggested approach:

   May be completed as a group, or a pair of consumers could focus on one activity and report back to group.

5. Questions and Feedback. Repeat from previous week

Week 5

Equipment: P2P workbook, post its/ butchers’ paper, pens/pencils/markers

1. Overview of the session: Repeat from previous week. (5 minutes)

2. Complete “Preparing for changes in activity participation” (Worksheet 5.1) while facilitating further discussion. Consumers answer 7 brief questions relating to activities in which they currently participate focusing on meaning and enjoyment. (20 minutes)

3. Complete “Prioritising plans for activity change” (Worksheet 5.2) while facilitating group discussion: Consumer returns to the “Activity Engagement Measure” (Worksheet 2.6) they completed in Week 2. They will identify 1-3 areas they would most like to change and specific activities they would like to experiment with. (25 minutes)

   If Worksheet 2.6 was not completed previously, ask consumers to rate their current satisfaction in these areas.

   TEA BREAK. (15 minutes)

4. Complete “Planning for activity change” (Worksheet 5.3): Leading on from the previous activity, consumers consider challenges that could arise from making changes to these activities. Facilitate a group discussion where consumers share examples and develop strategies for overcoming these. Consumers then complete “My plan for activity change” (Worksheet 5.4) where they plan their change experiment for the coming week. (30 minutes)

5. Discuss homework for the coming week "Thinking about your week" (Worksheet 5.5): Ask consumers to spent 5 minutes at the end of each day reflecting on their activities, and completing the homework worksheet ("Record of Activity Experiments") to identify positive and negative aspects of the day and their overall feeling. (10 minutes)

6. Questions and Feedback. Repeat from previous week

Week 6

Equipment: P2P workbook, post its/ butchers' paper, pens/pencils/markers

1. Overview of the session: Repeat from previous week. (5 minutes)

2. Group discussion around barriers to participation. Now consumers have attempted some change experiments, they may encounter some unexpected barriers. Complete "What stops me from moving on?" (Worksheet 6.1): Consumers can use the list provided and add their own examples that specifically relate to their circumstances. (45 minutes)

   Suggested approach:

   The Person/Environment/Occupation model may provide a useful structure and encourage consumers to contribute their own examples.

   TEA BREAK. (15 minutes)

3. Review of Change Experiments. Ask consumers who feel comfortable sharing to talk about their change experiment from last week and encourage group discussion of what went well and how to overcome any issues encountered. It important to ensure this discussion is supportive and encouraging, while also providing constructive feedback for improvement. Consumers can use the homework task from last week to reflect on how their experiment made them feel. (35 minutes)

4. Consumers then complete "My plan for activity change" (Worksheet 6.2) where they outline 2-3 further change experiment/s for the coming week. They are also asked to continue completing the homework worksheet ("Record of Activity Experiments"). (10 minutes)

5. Questions and Feedback. Repeat from previous week

Week 7

<u>Equipment:</u> P2P workbook, post its/ butchers' paper, pens/pencils/markers, 'What work means to me' cards

1. Overview of the session: Repeat from previous week. (5 minutes)

2. Group discussion of changes in participation patterns as a precursor to employment or other productive activities. Complete "Identifying changes in my activity patterns" (Worksheet 7.1): Consumers will revisit the areas they wanted to work on from week 2 (Worksheet 2.6) and week 5 (Worksheet 5.2) and identify the progress they have made toward these over the past five weeks. Consumers will be encouraged to discuss their recent activity experiments, reflecting on what went well and what they would like to work on in more detail. (45 minutes)

TEA BREAK. (15 minutes)

3. Group discussion about vocational / productive activities. Emphasise that participation in these activities is far more than paid employment and brainstorm the full range of options.

4. Complete "Reflecting about changes in my activity patterns" (Worksheet 7.2): Consumers will answer the questions based on their recent changes in activity. Once complete, facilitate a group discussion to identify challenges and the strategies consumers have used to overcome these. (20 minutes)

5. Complete "What Work Means to Me" activity: Consumers receive a set of cards each and map them into a diamond (most important factor on top, least important factor on bottom). Facilitate group discussion around the variation in how people arrange these factors, and ask consumers to copy their diamond onto 'What Work Means to Me" (Worksheet 7.3).

6. Introduce Homework: "My energy levels". Consumers will monitor their energy levels through the day and score these retrospectively every hour (2= energised, 1= aware but tired, 0= sleeping). The questions on the sheet will be completed in the next session.

7. Questions and Feedback. Repeat from previous week

Week 8

<u>Equipment:</u> P2P workbook, post its/ butchers' paper, pens/pencils/markers

1. Overview of the session: Repeat from previous week. (5 minutes)

2. Group discussion of "My energy levels" homework. What times of the day were people most energised? What times of the day were people most feeling tired? What strategies do the consumer use to manage fatigue?

   Prompt question: How would you stay energised for a long day? Place all ideas on butchers' paper and encourage consumers to copy those of interest in the workbook. (40 minutes)

TEA BREAK. (15 minutes)

3. Complete “What Stops Me from Moving On” (Worksheet 8.1): Consumers are encouraged to think about barriers specific to vocational / productive activities. Facilitate a group discussion about the barriers specific to these activities and strategies for overcoming them. (25 minutes)

4. Group discussion of “work/life balance” – What is this concept about? Why is it important? How would vocational / productive activities fit into your current day? Complete the “Weekly Planner” (Worksheet 8.2): Consumers are to fill this in with their current activities and identify times / days when they could undertake vocational / productive activities. (20 minutes)

5. Introduce Homework “What do you like or enjoy”: Ask consumers to complete this homework sheet during the coming week in preparation for next week’s activities.

6. Questions and Feedback. Repeat from previous week

Week 9

Equipment: P2P workbook, post its/ butchers’ paper, pens/pencils/markers

1. Overview of the session: Repeat from previous week. (5 minutes)

2. Complete “Doing and Skills” (Worksheet 9.1) while facilitating a group discussion: Consumers use activities in a typical day to identify transferable skills they already possess (e.g., contacting a friend by phone involves the ability to use a phone, being able to verbally communicate and perhaps form rapport). Discuss the spread of skills and qualities possessed by the group and write examples on the board or butchers’ paper to show the ‘capital’ available in the group. (25 minutes)

3. Complete “What have I done” (Worksheet 9.2): Following on from the previous activity, consumers will consider all the activities (i.e. beyond those in a typical day) they have experienced and use these to identify further transferable skills (20 minutes)

TEA BREAK. (15 minutes)

4. Complete “Things to write on my resume” (Worksheet 9.3): Based on the previous two activities and last week’s homework, consumers will identify five potential skills and/or qualities they could include in a resume including an example when they have demonstrated this (20 minutes).

5. Complete “Self Profile” (Worksheet 9.4): Consumers complete the self-profile again without reference to the first one (Worksheet 1.1). They will then compare the two (week 1 and today), reflecting on any changes they have made. Facilitate a group discussion about these changes highlighting one specific achievement for each consumer. (25 minutes)

6. Questions and Feedback. Repeat from previous week

Week 10

Equipment: P2P workbook, post its/ butchers' paper, pens/pencils/markers

1. Overview of the session: Repeat from previous week. (5 minutes)

2. Introduce the guest speaker (Vocational consultant from local employment agency): Ideally their presentation will combine information about local opportunities for both paid and unpaid employment, with question-and-answer elements or informal discussion. (40 minutes)

TEA BREAK. (15 minutes)

3. Complete "Reflecting on our goals" (Worksheet 10.1): Consumer revisit the goals they set in the first week and assess whether they met them or not. Facilitate a group discussion around this, and support consumers to set new goals for the next three months. (30 minutes)

4. Graduation: Present each consumer with their certificate of completion and formally conclude the program. (20 minutes)

5. Questions and Feedback. Repeat from previous week
